# Pediatric Surgical Volume at the TASH: Experience after the Expansion of Pediatric Surgery Program

**DOI:** 10.4314/ejhs.v31i6.14

**Published:** 2021-11

**Authors:** Samuel Negash, Workiye Tigabe, Woubedel Kiflu, Miliard Derbew

**Affiliations:** 1 Division of Pediatric Surgery, Department of Surgery, Addis Ababa University, Ethiopia

**Keywords:** Global surgery, Pediatric surgery, Surgical training, morbidity and mortality

## Abstract

**Background:**

Ethiopia has a high unmet need for pediatric surgical conditions. Over the past 5 years, new changes have been introduced to the pediatric surgery division at Addis Ababa University to overcome this issue. The changes include initiation of pediatric surgery residency, allocating operating room for pediatric surgery, weekend surgical campaign and starting ultrasound guided hydrostatic reduction. We conducted this study to evaluate the pattern and outcome of pediatric surgical cases after these changes.

**Methods:**

The study was a retrospective review conducted at Tikur Anbessa Hospital from Jan – Dec 2019. Data was collected from duty report forms that included emergency procedures, admissions and mortalities. Data on elective procedures was collected from operation theater log books.

**Results:**

Overall, a total of 1590 pediatric surgical procedures were performed during 2019 of which 942 cases were elective and 648 were emergency. This was an increment in number of surgeries performed by 75%. The leading emergency procedure was aerodigestive foreign body removal which increased by 46%. Surgery for intussusception has decreased by 30% with increasing use of hydrostatic reduction. Overall, average morbidity and mortality was 3.5% and 6.9% respectively. Morbidity and mortality rates were similar throughout the year.

**Conclusion:**

The study shows increased productivity over the past year with the changes made in the department. There is also no increment in morbidity and mortality during the start of the academic year. This implies adequate consultant supervision of residents during transition.

## Introduction

Lack of surgical care has become a major cause of morbidity and mortality with significant implications for public health (1–3). The situation is magnified in children of African countries where there is severe limitation in access to surgical care (4). An important challenge is the lack of trained health care providers (5). There are only 52 pediatric surgeons in the college of surgeons of east, central and southern Africa. Of these, only 11 are in Ethiopia, a country with a population of 102.4 million (6).

To address this critical shortage, a 5-year pediatric surgery residency program was introduced at Addis Ababa university, Ethiopia. Pediatric surgical training was supervised by 7 consultant surgeons. The first 8 residents joined the program in January 2018 and the second batch joined in 2019 to make a total of 17 residents. Literatures have shown different impacts of residency training. Some state an increase in hospital case volume and complexity, which is advantageous in increasing surgical access (7). Others point out negative patient outcomes due to quality of care delivered by residents (the “July effect”) (8,9).

Additional modification made at our hospital was to have additional access to operating room. This was done by reallocating operating room that used to be utilized by another surgical specialty in 2016. Furthermore, funding was raised to conduct a surgery campaign during the 3^rd^ quarter of 2019. For this purpose, two operating rooms were allotted for use during the weekends.

Another change was the practice of ultrasound guided hydrostatic reduction for intussusception. The program started in in 2014 and the pilot study on 53 patients showed high success of 77% (10). However, surgery for intussusception still remained one of the commonly performed procedures the next 3 years (11).

So far, two studies have been conducted in our hospital entailing the overall pattern of pediatric surgical admissions and procedures (11,12). However, we wanted to reassess the impact of the new changes on case volume, case complexity and outcome of patients.

## Patients and Methods

This was a cross-sectional study performed in Tikur Anbessa Specialized Hospital, Addis Ababa, Ethiopia. This is the largest referral hospital in the country serving millions of people. The unit of pediatric surgery in our hospital was established in 2009. Since 2016, the unit has 7 consultant surgeons and performs around 1000 procedures per year. The scope of practice includes neonatal, oncologic, gastrointestinal, colorectal, urology and non-cardiac thoracic surgeries.

All general pediatric surgery patients (<13 years) who underwent surgery from January to December 2019 were included in the study. Patients for whom ultrasound guided hydrostatic reduction was done for intussusception were also included.

Data was collected retrospectively from daily duty report forms, operating room registries and monthly audit reports. Variables included were diagnosis, type of procedure, date of procedure, morbidity and mortality. Data entry and analysis was done using SPSS version 25. Data was compared with previous studies from this hospital. Ethical approval was obtained from ethics and research committee at department of surgery, college of health sciences Addis Ababa University.

## Results

Overall, a total of 1590 pediatric surgical procedures were performed during 2019. All cases were included in the study. Of these 942 (59%) were elective and 648 (41%) were emergency. The top 10 elective and emergency procedures are described in Table 1.

**Table 1:** Commonly performed surgical procedures in the unit of pediatric surgery at Tikur Anbessa Hospita in 2019

	Elective	N (%)	Emergency	N (%)
1	Hypospadias repair	90 (9.6%)	Bronchoscopic FB removal	140 (21.6%)
2	Cystoscopy	90 (9.6%)	Colostomy/ileostomy	104 (16.0%)
3	Orchiopexy	74 (7.6%)	Esophageal FB removal	94 (14.5%)
4	PSARP / ASARP	72 (7.6%)	Surgery for EA	36 (5.55%)
5	PPV ligation	58 (6.2%)	pyloromyotomy	28 (4.3%)
6	Stoma reversal	55 (5.8%)	Surgery for intussusception	26 (4%)
7	Pullthrough	49 (5.2%)	Appendectomy	25 (3.9%)
8	Rectal biopsy	33 (3.5%)	Intestinal anastomosis for atresia	23 (3.5%)
9	UCF repair	22 (2.3%)	Chest tube insertion	23 (3.5%)
10	Colostomy	20 (2.1%)	I & D of abscess	20(3.1%)

PSARP = posterior sagittal anorectoplasty, ASARP = anterior sagittal anorectoplasty, PPV = persistent process vaginalis, UCF = urethrocutaneous fistula, EA = esophageal atresia, I&D = incision and drainage

Pediatric urology procedures were the most common elective procedures in our setup. These included surgery for hypospadias (9.6 %), cystoscopy (9.6%), undescended testes (7.6%), inguinal hernia/hydrocele (6.0%) and repair of urethrocutaneous fistula (2.3%). These were followed by colorectal procedures, mainly surgery for anorectal malformation and Hirschsprung disease. Procedures performed for these conditions include stoma creation (2.1%), stoma reversal (5.5%), rectal biopsy (3.5%), pullthrough (5.2%) and anorectoplasty (7.6%).

The most common emergency procedures were foreign body removal (esophagus and bronchoscopy) which combined accounted for 36% of emergency procedures. This was followed by neonatal surgical procedures (13.5%) which included surgery for hypertrophic pyloric stenosis, esophageal atresia, and intestinal atresia. Colostomy/ileostomy (16%) was also one of the top emergency procedures mainly performed in neonates. Other emergency surgical procedures include surgery for appendicitis, intussusception, chest tube insertion and Incision-drainage of abscesses. Surgery was performed for 26 patients with intussusception while hydrostatic reduction was performed for 89. Surgery for intussusception accounted for 4% of emergency procedures.

**Figure 1:**
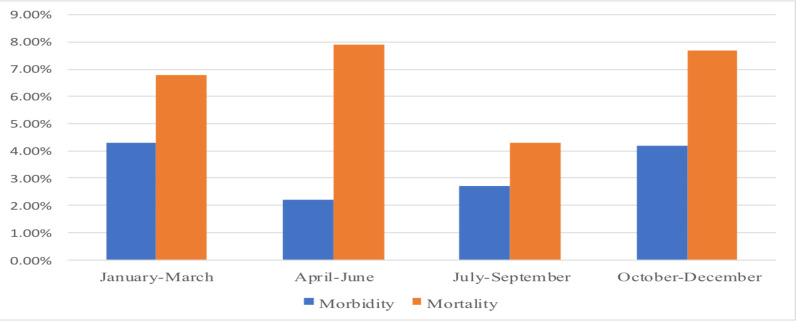
Trend of morbidity and mortality rate in the unit of pediatric surgery at Tikur Anbessa Hospital in 2019

The morbidity ranged from 2.2 – 4.3 % with average major morbidity 3.5%. Mortality ranged from 4.3–7.9% with year average of 6.9%. The most common causes of mortality were emergency neonatal surgical conditions. These included esophageal atresia (2.4%), intestinal atresia (1.2%), abdominal wall defects (0.7%), anorectal malformation (0.6%), Hirschsprung disease (0.3%) and hypertrophic pyloric stenosis (0.2%). Common causes of mortality in older children were emergency procedures including foreign body aspiration (0.4%) and intussusception (0.3%) (Table 2). There was no significant difference in morbidity and mortality throughout the academic year (Figure 1).

**Table 2:** causes of morbidity and mortality in pediatric surgical patients at Tikur Anbessa Hospital in 2019

Morbidity	N (%)	Mortality	N (%)
Superficial SSI	17 (1%)	EA/TEF	38 (2.4%)
Failed/incomplete FB removal	10 (0.6%)	Intestinal Atresia	19 (1.2%)
		Gastroschisis/ omphalocele	11 (0.7%)
Anastomotic leak	8 (0.5%)	Anorectal malformation	10 (0.6%)
pneumonia	6 (0.4%)	FB aspiration	6 (0.4%)
Deep SSI (abscess)	4 (0.3%)	Intussusception	4 (0.3%)
Parastomal hernia	2 (0.1%)	HD	4 (0.3%)
Other	8 (0.5%)	IHPS	3 (0.2%)
Total	55 (3.5%)	Other	15 (0.9%)
		Total	110 (6.9%)

SSI = surgical site infection, EA/TEF = esophageal atresia, tracheoesophageal fistula, FB = foreign body, HD = Hirschsprung disease, IHPS = idiopathic hypertrophic pyloric stenosis

## Discussion

**Figure 2:**
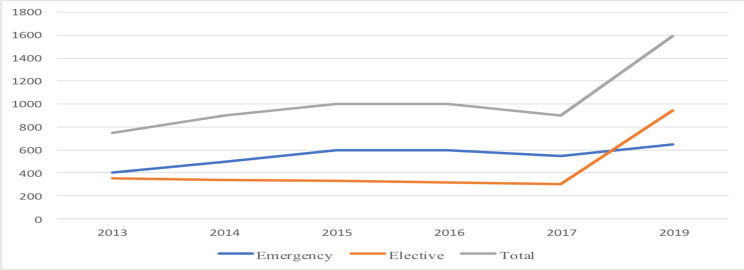
Trend of number of pediatric surgical procedures performed yearly in Tikur Anbessa Hospital

Overall, a total of 1590 pediatric surgical procedures were performed during 2019. We found a significant increase in case volume by 75% compared to previous reports. Major spike was in the elective activities which showed increment by 160%. Emergency procedures were relatively stable with increase of 18% (11). (Figure 2) These figures indicate increased efficiency enabling us to operate more patients from the waiting list. The finding is more impressive as no additional staff was added during the study period. We attribute this success combined effect of allocating additional operating rooms and weekend surgical campaigns. The efficiency of attendings working with the help of new residents may also have a contribution. Another study has also shown initiation of training program can increase case volume even in the absence of additional operating room access (7).

Proportion of pediatric surgical conditions was similar to previous reports from Ethiopia as well as other African countries (2,11–14). We found congenital anomalies are still the most prevalent indication for elective surgeries. Based on organ system affected, urologic anomalies are the most common followed by colorectal. Further specialization in these areas may be considered to improve the quality of care.

Emergency procedures also have a similar pattern with aerodigestive foreign bodies being the most common indication for surgery (13). Previous reports have shown consistently increasing number of aerodigestive FB, with recent increment possibly related to the introduction of a new coin in our country (11,12,15,16). It has been suggested that availing bronchoscopy facilities in other parts of the country might alleviate the case burden in our institution (16). Another institution has also reported using flexible endoscopy to manage these children (17). We found aerodigestive foreign body has increased by 46% from our previous study (11). This shows the growing problem still remains. We believe more effort is required in public education and prevention than focusing on the management these patients.

Another impact was on the management of intussusception. Our hospital was the first in the country to initiate hydrostatic reduction for intussusception (10). A previous study which included the first 3 years after initiation of this procedure did not find significant difference in the management of these patients (11). However, this study has shown surgical management of intussusception has decreased to 4% of emergency pediatric surgical procedures (previously 7%). Yearly average number of surgeries for intussusception also dropped by 30% (from 37 to 26) (11). In addition, the number of hydrostatic reduction has increased by 68% (from 53 to 89) (10). Overall trend shows success in avoiding surgery with associated cost and morbidity.

Morbidity rates from this report may be lower as many mild complications may not be recorded in charts and will be missed in a retrospective review. Mortality rates in our center varies from 2% to 58%. This is because previous mortality reports only included a specific subset of patients like neonatal procedures and emergency conditions (13). This study looks at the overall mortality in our institution which was 6.9%. This is comparable to past studies from sub-Saharan Africa with reports of 5.3% from Gambia (18) and 5% from Tanzania (2). A recent systematic review of pediatric surgery in African LMIC found a higher mortality of 12% (19).

Finally, we also wanted to evaluate the impact of residency training on patient outcome. Some studies have suggested increased morbidity and mortality at the start of the academic year with residents' transition (9). There was a slightly decreased mortality in the 3^rd^ quarter of the year (July-September) which we attributed to the surgical campaign conducted during that period. Otherwise, we found no significant difference in patient outcome between the earlier and later periods of the academic year. This indicates good monitoring and support of the trainees by consultants.

In conclusion, there is an increased case volume without adding additional staff which indicates improved productivity with start of the training program and other changes made to the unit. Further specialization in pediatric urology and colorectal surgery should be considered, as these are the most prevalent conditions in our setup. Surgery for intussusception has significantly declined with the increasing utilization of hydrostatic reduction. This is an encouraging finding that should prompt the expansion of this practice to other hospitals all over the country. The trend of aerodigestive foreign bodies is alarming. Future directions should be geared towards prevention by working together with the public health department. We also recommend to have further prospective studies in each of these areas to implement quality improvement projects.
